# Decoding the role of TET family dioxygenases in lineage specification

**DOI:** 10.1186/s13072-018-0228-7

**Published:** 2018-10-05

**Authors:** Xinwei Wu, Gang Li, Ruiyu Xie

**Affiliations:** Centre of Reproduction, Development & Aging, Faculty of Health Sciences, University of Macau, Macau SAR, 999078 China

**Keywords:** Lineage specification, TET, 5hmC, 5mC, Bivalent promoter, Enhancer

## Abstract

Since the discovery of methylcytosine oxidase ten-eleven translocation (TET) proteins, we have witnessed an exponential increase in studies examining their roles in epigenetic regulation. TET family proteins catalyze the sequential oxidation of 5-methylcytosine (5mC) to oxidized methylcytosines including 5-hydroxymethylcytosine (5hmC), 5-formylcytosine, and 5-carboxylcytosine. TETs contribute to the regulation of lineage-specific gene expression via modulating DNA 5mC/5hmC balances at the proximal and distal regulatory elements of cell identity genes, and therefore enhance chromatin accessibility and gene transcription. Emerging evidence suggests that TET dioxygenases participate in the establishment and/or maintenance of hypomethylated bivalent domains at multiple differentiation-associated genes, and thus ensure developmental plasticity. Here, we review the current state of knowledge concerning TET family proteins, DNA hydroxymethylation, their distribution, and function in endoderm, mesoderm, and neuroectoderm specification. We will summarize the evidence pertaining to their crucial regulatory roles in lineage commitment and development.

## Background

DNA methylation and the recently identified hydroxymethylation are essential epigenetic modifications in cells. DNA methylation is catalyzed by DNA cytosine-5-methyltransferases (DNMTs) via transferring a methyl group to the 5′ position of cytosine to form 5-methylcytosine (5mC). Heavily methylated DNA is often associated with repressed gene expression. In addition, DNA methylation can be actively demethylated by DNA methylcytosine dioxygenases, the ten-eleven translocation (TET) proteins, through oxidizing 5mC into 5-hydroxymethylcytosine (5hmC) and further to 5-formylcytosine (5fC) and then to 5-carboxylcytosine (5caC). Finally, 5fC and 5caC are removed by thymine DNA glycosylase (TDG) and cytosine is replaced by base excision repair (BER) [1–5]. Successful oxidation of 5mC by TET proteins influences various biological properties such as chromatin accessibility, nucleosome positioning, genomic stability, and rates of gene transcription [6–9]. TET proteins hence serve as important epigenetic modifiers that participate in a number of biological processes including embryogenesis, lineage specification, and disease development. Herein, we specifically review how TET proteins and their enzymatic products contribute to the regulation of cell lineage commitment and development.

## Structural basis of TET family proteins

TET proteins are widely expressed in various organisms including human, mouse, Xenopus, and zebrafish [6, 10–14]. The three TET family members (TET1, TET2, and TET3) share a highly conserved catalytic domain at the C-termini, which comprises cysteine-rich and double-stranded β-helix (DSBH) regions [15, 16]. The DSBH region contains ferrous (Fe^2+^) and α-ketoglutarate (α-KG) binding sites that are critical for TET catalytic activity [17, 18]. TET1 and TET3 both possess a zinc finger cysteine-X-X-cysteine (CXXC) domain at the N-termini, which allows them to bind to cytosine and its modified forms (e.g., 5mC, 5hmC, 5fC, and 5caC) in DNA [6, 19, 20]. Although TET2 does not encode the CXXC domain, it targets deoxynucleotides through another CXXC-containing protein known as IDAX or CXXC4. Therefore, TET2 appears to bind to DNA in a similar fashion as TET1 and TET3 [21]. Recently, a CXXC-domain deficient short form of TET1 (TET1S) has been identified in both mouse and human somatic cells [22, 23]. TET1S retains reduced catalytic activity of TET dioxygenase and displays a weaker binding affinity for DNA [22]. Despite their differences in DNA binding properties, these TET isoforms are comparable in terms of 5mC oxidation capability.

## Dynamic distribution of TETs and 5hmC during development

The three TET family members have distinct expression patterns among different cell types and are tightly regulated during development [24–26]. TET1 protein is highly expressed in both human and mouse embryonic stem cells (ESCs), whereas TET2 is expressed at extremely low levels in human ESCs similar to TET3 in mouse ESCs [26–28]. The expression levels of TET proteins change dynamically during development. For instance, TET3 is expressed at high levels in oocytes and zygotes, and undergoes rapid downregulation in two-cell-stage embryos [29]. It has been shown that TET3 expression increases dramatically in human ESC-derived neuroectoderm and pancreatic endoderm [24, 28], which is consistent with its progressive increase in mouse embryos from e6.5 to e9.5 [30]. In contrast, TET2 is widely expressed in a variety of somatic organs and cell types, especially in hematopoietic cells [31]. More interestingly, recent studies have illustrated that two TET1 isoforms also display distinct expression patterns, in which the full-length isoforms of TET1 are preferentially expressed in ESCs, early embryos, and primordial germ cells (PGC), while the short form of TET1 (TET1S) is restricted to somite cells and overexpressed in cancer [22, 23, 32]. An isoform switch of TET1 has been implicated in influencing gametic imprinting, PGC development, and epigenetic memory erasure [22].

Since the discovery of TET family dioxygenases, studies examining the function of DNA hydroxymethylation have exponentially increased. Emerging evidence indicates that 5hmCs are enriched at low-to-intermediate CpG density regions of promoters and enhancers of developmental regulatory genes [33–42]. Similar to TET family proteins, the distribution of 5hmC is dynamically changed and positively correlated with active gene transcription during lineage specification [35, 43, 44]. For example, high levels of 5hmC are found in ESCs and in the central nervous system [16, 45]. Global 5hmC levels decrease during ESCs differentiation toward neuroectoderm fate, while enrichment of 5hmC at the gene body of transcriptionally active genes is identified in neural progenitor cells (NPCs) [43, 44]. When NPCs further differentiate into neurons, overall 5hmC increases are accompanied by a loss of H3K27me3 at promoters of genes that are important for neuronal function [34]. Likewise, a human ESC-based model of pancreatic differentiation reveals that global 5hmC levels rapidly decrease at the first step toward definitive endoderm, then gradually increase toward pancreatic endoderm specification. 5hmC enriching peaks significantly overlap with poised and active enhancers, as well as the boundaries of hypomethylated functional genomic regions [28]. Furthermore, enrichment of 5hmC at tissue-specific enhancers has also been demonstrated in cardiomyocyte and hematopoietic cell differentiation [10, 35, 46]. Other than at promoters and enhancers, enrichment of 5hmC is also found over the gene bodies of actively transcribed genes [35, 36, 47]. Taken together, these studies suggest that expression of TET family proteins and distribution of 5hmC is differentially regulated to meet the needs of cellular functions during lineage commitment.

## Mechanisms of TET family proteins in the regulation of gene transcription

The establishment of cellular identities requires precise control of gene expression. Mounting evidence suggests that TET proteins work as epigenetic players to alter DNA methylation, histone modification, and chromatin accessibility, which regulate the transcription of key developmental genes. Strikingly, promoters, enhancers, and DNase I-hypersensitive sites accumulate significantly more 5mC in *Tet1*, *Tet2*, and *Tet3* triple knockout mouse ESCs, suggesting that these gene regulatory regions are the major targets of TETs [13, 48, 49]. In the following subsections, we discuss the current mechanistic understanding of how TET proteins regulate lineage-specific gene expression.

### Maintenance of hypomethylated promoters of developmental genes

Accumulated evidence indicates that DNA methylation status at promoter regions influences gene transcription [50]. Promoter hypermethylation is believed to contribute to the establishment of a transcriptionally poised/inactive state [50]. For example, pluripotent genes, such as *OCT4*, are actively expressed in human ESCs and suppressed by promoter hypermethylation upon differentiation [44]. It has been widely documented that genetic ablation of *TET* induces promoter hypermethylation and aberrant gene expression in multiple lineage differentiation systems [6, 25, 27, 38, 51–53]. For example, TET2 is required for the maintenance of NANOG expression in the spontaneous differentiation of mesodermal lineage cells from human ESCs. TET2 ChIP-seq revealed that TET2 associated with the *NANOG* promoter prevents DNA methylation [27]. In helper T cell differentiation, Tet2 is recruited to the promoters of cytokine genes in a lineage-specific transcriptional factor-dependent manner, which stimulates active DNA demethylation and expression of these cytokine genes [38]. TET1 and TET3 also regulate DNA methylation status in the promoter regions. For instance, Tet3 directly binds to the promoters of genes critical for neural development in *Xenopus*, such as *Pax6*, *Rx*, and *Ngn2*, and sustains high levels of 5hmC at promoters [6]. Furthermore, simultaneous deletion of *Tet2* and *Tet3* downregulates *P2rX7* expression along with reduction of 5hmC at the *P2rX7* promoter during bone marrow mesenchymal stem cell differentiation [53], indicating that TET-mediated rapid and specific oxidation of 5mC at promoter loci is biologically relevant.

**Fig. 1:**
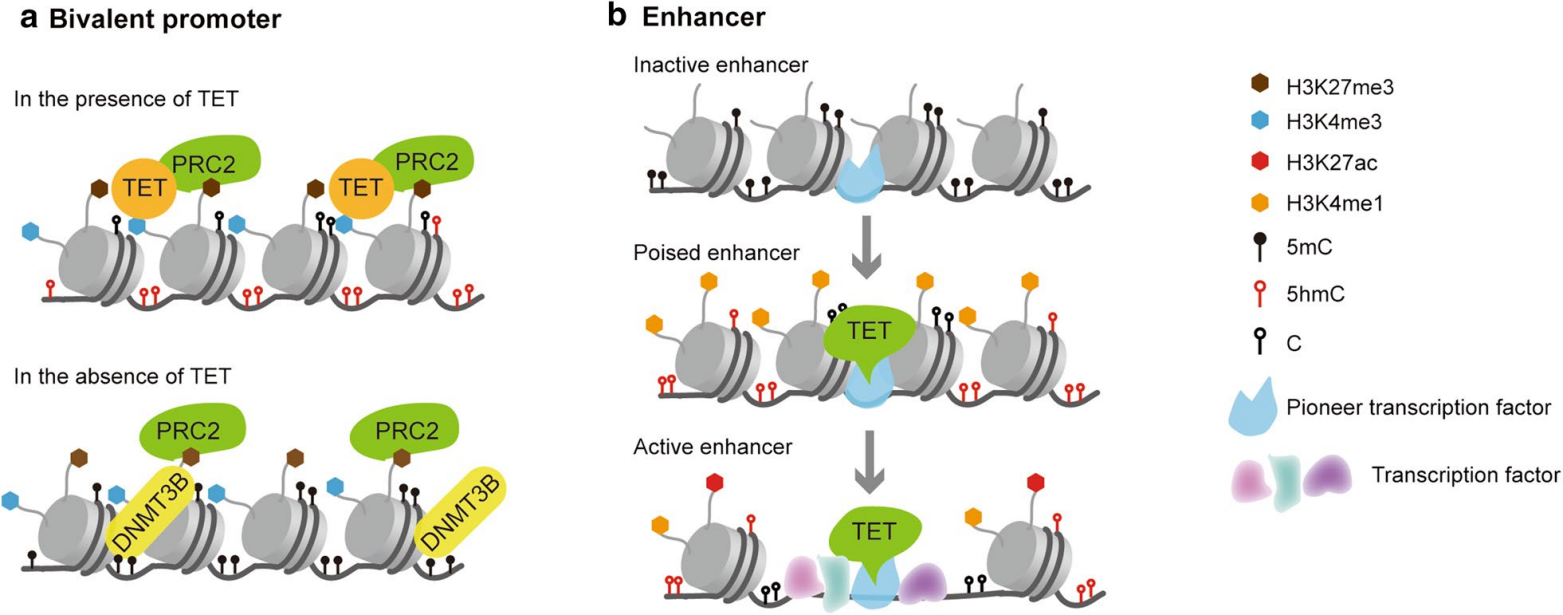
The role of TET proteins on lineage-specific bivalent promoters and enhancers. **a** In the presence of TET dioxygenases, PRC2 recruits TETs to bivalent promoters to maintain their hypomethylated status. In the absence of TETs, binding of DNMT3B at the bivalent promoters causes de novo DNA methylation, which leads to stable gene silencing and loss of developmental plasticity. **b** A model of TET-mediated enhancer priming and activation. Upon differentiation, pioneer transcription factors that are not sensitive to DNA modifications can bind to distal enhancers of lineage-specific genes and recruit TETs to demethylate methylcytosines. Other epigenetic modifiers, such as p300 and SET1/COMPASS, subsequently bind to these sites and establish poised (H3K4me1) and active (H3K27ac) enhancers, which in turn increases chromatin accessibility and allow other transcription factors binding to occur

More interestingly, TET-mediated DNA demethylation has been suggested in association with the establishment and/or maintenance of bivalent promoters of developmental genes, in which H3K4me3 and H3K27me3 histone modifications take place simultaneously [14, 54]. In general, bivalent promoters of developmental genes are hypomethylated in ESCs [55]. They are preferentially repressed by trimethylation of histone H3 at lysine 27, which is easier to be reversed than DNA methylation. These low-methylated genetic loci can extend beyond promoter regions, forming H3K27me3-marked DNA methylation valleys (DMVs) that provide binding sites for a large set of transcription factors to mediate complex regulation during development [56]. It has been recently shown that TET proteins and Polycomb Repressive Complex 2 (PRC2), which is responsible for H3K27 methylation, can recruit each other to maintain a hypomethylated status at bivalent promoters and DMVs [57–59]. In addition, TETs can interact with OGT (O-linked *β*-*N*-acetylglucosamine transferase), which enhances methylation of histone H3 at lysine 4 by promoting the binding of a component of the H3K4 methyltransferase SET1/COMPASS complex to active promoters [60]. It has been further demonstrated that overexpression of TET2, but not the catalytically inactive TET2, results in an increase in 5hmC at a particular set of key developmental gene promoters, which is sufficient to promote DNA demethylation and de novo bivalent modifications [54]. This discovery was subsequently supported by a recent report which illustrated that TETs safeguard bivalent promoters of many lineage determinants, such as *FOXA2*, *GATA2*, *PAX6*, and *SOX17*, by preventing their aberrant hypermethylation to ensure developmental competency [14]. Together, these studies suggest that TET-mediated DNA demethylation retains developmental plasticity at the promoters and/or DMVs of developmentally important genes, and therefore ensure robust induction of lineage-specific transcription upon differentiation (Fig. 1).

### Regulation of chromatin accessibility and enhancer architecture

In addition to facilitate promoter hypomethylation, TET proteins also play critical roles in regulating chromatin accessibility and enhancer architecture (Fig. 1). 5hmC co-localizes with enhancers and open chromosome regions during different biological processes, such as B cell differentiation and pancreatic endocrine differentiation [7, 28, 61]. By examining genome-wide DNA methylation and hydroxymethylation in the context of *Tet2* deletion in mESCs, Ren and colleagues have found that depletion of *Tet2* leads to enhancer hypermethylation, accompanied by the loss of active enhancer mark H3K27ac and delayed the induction of *Slit3*, *Lmo4*, and *Irx3* upon differentiation to a neural progenitor fate [62]. A similar observation has been made in cardiomyocytes where deletion of *Tet2* causes loss of 5hmC at enhancers and is accompanied by extensive elevation of DNA methylation, reduction of H3K27ac, and impaired gene expression during heart development [10]. Re-expression of Tet2 catalytic domain in *Tet2/3* double knockout pro-B cells restores chromatin accessibility at a genome-wide level as well as at the *Igκ* enhancer [7]. In agreement with the above studies, application of TET inhibitor dimethyloxalylglycine (DMOG) reduces chromatin accessibility at specific enhancers of P19 embryonic carcinoma cells when differentiated to NPCs [63]. These data implicate the functional relevance of TETs and TET-mediated 5hmC to chromatin accessibility at distal regulatory elements, particularly enhancers. Although the precise molecular mechanisms remain unclear, epigenetic readers of 5hmC, such as MeCP2 (methyl-CpG-binding protein 2), might constitute a mechanism of TET-regulated chromatin opening [64].

In addition to chromatin accessibility, TETs may also contribute to enhancer priming. Prior to activation, the enhancer region is methylated and not accessible to general transcription factors. However, pioneer transcription factors, such as FOXA1, MEIS1, and PBX1, preferentially bind to oligonucleotide probes which contain methylated cytosines [63, 65]. It has also been suggested that pioneer transcription factors can physically interact with TETs which catalyze oxidation of 5mC at gene regulatory regions [7, 66, 67]. For example, pioneer transcription factors, PU.1, recruit Tet2 proteins to the *Igκ* enhancer to facilitate DNA demethylation during early B cell maturation [7]. Furthermore, removal of 5mC or deposition of 5hmC coincides with increased accessibility of enhancers and monomethylation of H3K4 [12, 28, 63]. Accordingly, H3K4 methyltransferases are repelled by 5mC [68, 69], indicating that TET-mediated DNA demethylation is necessary for the recruitment of H3K4 methyltransferases to prime enhancers. Additionally, it has been demonstrated that TETs can recruit SET1/COMPASS H3K4 methyltransferase as well as histone acetyltransferase p300 to gene regulatory regions [38, 60]. Therefore, H3K4 monomethyltransferases MLL3/4, which are COMPASS family members, might also interact with TETs and become recruited to enhancer regions [70].

Despite their plausible contribution to chromatin architecture, TETs may regulate gene transcription by interfering with RNA polymerization and RNA splicing. In CD4^+^ T cells, TET1 and TET2 alter CTCF-dependent alternative pre-mRNA splicing through oxidation of 5mC to 5hmC and 5caC at corresponding intragenic CTCF-binding sites in the *PTPRC* (protein tyrosine phosphatase CD45) locus [71, 72]. Additionally, it has been suggested that oxidation derivatives of 5hmC are concentrated on the gene bodies of transcribed genes and support transcriptional consistency [35, 47].

## TET dioxygenases modulate cell fate commitment

Growing evidence confirms that TET family proteins are important regulators for embryogenesis. *Tet1/2/3* triple knockout mice are embryonic lethal with severe gastrulation defects during embryogenesis [13, 30, 48]. Although the formation of three germ layers is initiated, Tet-null mouse embryos cannot further develop, accompanied by impaired patterning of axial mesoderm, neuroectoderm, and definitive endoderm [13, 30]. Furthermore, knockdown of *TET2* skews spontaneous differentiation of human ESCs into neuroectoderm with the loss of mesoderm and endoderm [27], while inactivation of Tet2 in mouse hematopoietic stem cells leads to an increase in the granulocytic and monocytic population [31]. Thus, deficiency of TET proteins disturbs the 5mC and 5hmC landscapes, which causes differentiation to switch from one to another lineage [30, 61, 73, 74]. These studies illustrate the critical function of TET family proteins in development. Below, we will discuss the roles of TET proteins in each lineage commitment (Table 1).

**Table 1:** Phenotypes resulting from the depletion of *TETs* in ectoderm, mesoderm, and endoderm lineages

Lineage	System	TET isoform	Phenotype	References
Ectoderm	Human ESCs	*TET1/2/3 triple* knockout	Form fewer PAX6^+^ neuroectoderm cells	[14]
	Neuron stem cells	*Tet1* or *Tet2* knockdown	Reduce proliferation of neuron stem cells	[90]
	Cortex	*Tet2* or *Tet3* knockdown	Abnormal accumulation of cell clusters along the radial axis in the intermediate zone and ventricular zone	[34]
	Cerebellar granule cells	*Tet1/3* double knockdown	Impair dendritic arborization of cerebellar granule cells	[77]
	Mouse ESCs	*Tet3* knockout	Apoptosis of neuron progenitor cells and reduce terminal differentiated neurons	[24]
	Neurons	*Tet1* knockout	Increase hippocampal long-term depression and impair memory extinction	[75]
	Neurons	Tet1 overexpression	Promote neurogenesis	[91]
	Retinal neurons	*Tet2/3* double knockout	Defects in retinal cells terminal differentiation	[92]
	Cortical neurons	*Tet2* knockdown	Reduce neuronal cells survival	[93]
	Head	*Tet1* mutation	Defects in neural tube closure	[94]
	Eye and neural	*Tet3* knockdown	Eye malformations and small head	[6]
	Oligodendrocyte precursor cells	*Tet1*, *Tet2*, or *Tet3* knockdown	Reduce mature oligodendrocytes	[95]
	Olfactory sensory neurons	Tet3 overexpression	Disturb axon targeting and olfactory receptor expression	[47]
	Dental pulp cells	*TET1* knockdown	Prevent the proliferation and differentiation of dental pulp cells	[96]
Mesoderm	Hematopoietic stem cells	*Tet2* knockout	Enhance self-renewal of hematopoietic stem cells, expansion of myeloid progenitors	[97]
	Bone marrow cells	*Tet2* knockdown	Enhance self-renewal of hematopoietic stem cells, expansion of myeloid progenitors	[31]
	Hematopoietic stem cells	*Tet2/3* double knockout	Loss of hematopoietic stem cell-derived blood cells	[98]
	Human ESCs	*TET2* knockdown	Impair hematopoietic cell differentiation	[27]
	T cells	*Tet2/3* double knockout	Form more iNKT cell in the young mice, and skew major population to NKT17 cells	[74]
	Regulatory T cells	*Tet2/3* double knockout	Less regulatory T cells in the spleen and lymph nodes	[86]
	Regulatory T cells	*Tet1/2* double knockout	Less regulatory T cells in the spleen and lymph nodes	[83]
	T cells	*Tet2* knockout	Impair Th1 and Th17 cells differentiation and cytokine genes induction	[38]
	T cell	*Tet2* knockout	Promote memory CD8^+^ T cells differentiation after viral infection	[99]
	B cells	*Tet2/3* double knockout	Block progenitor B cells differentiation and maturation	[7, 61]
	Mast cells	*Tet2* knockout	Impair mast cell differentiation, cytokine production, and proliferation	[85]
	Erythroid cells	*TET2* or *TET3* knockdown	Delay differentiation of erythroid progenitors and regulate terminal differentiation	[84]
	Bone marrow mesenchymal stem cells	*Tet1/2* double knockout	Increase self-renewal of bone marrow mesenchymal stem cells and reduce osteogenic differentiation	[53]
	Smooth muscle cell	TET2 overexpression	Convert fibroblasts to smooth muscle cells	[51]
	Skeletal muscle myoblasts	*Tet2* knockdown	Impair myoblast differentiation	[88]
	Cardiomyocyte	*Tet2* knockdown	Downregulate genes related to cardiac muscle contraction and cardiac muscle fiber development	[10]
Endoderm	Intestinal stem cell	*Tet1* knockout	Form shorter intestine	[25]
	T84 colon adenocarcinoma cells	*Tet1* knockdown	Dysregulate genes related to cell membrane and extracellular space	[100]

### Neuroectoderm lineage specification

The brain is one of the places in mammals with the most abundant 5hmC, suggesting that TET proteins and TET-mediated 5hmC might have significant impacts in neurogenesis. *Tet1* knockout mice exhibit defects in neuron function including learning, memory consolidation, storage, and extinction [52, 75, 76]. Ablation of Tet1 leads to downregulation of neuronal activity-regulated genes such as *Npas4* [75]. Although Tet1 is highly expressed in multiple regions and cells of the brain like hippocampus, isocortex, and cerebellar granule cells [77, 78], *Tet1* knockout does not alter brain morphology. It has been revealed that compensatory upregulations of Tet2 and Tet3 were observed in the *Tet1* knockout mouse brain [76]. In addition, overexpression of Tet3 can facilitate the reprogramming of MEFs (mouse embryo fibroblasts) into neuronal cells, which is accompanied with an active demethylation process at the promoters of genes encoding neuron-specific transcription factors such *Ascl1*, *Brn2*, and *Ngn2* [79]. Therefore, TET family members are functionally redundant in neurogenesis. In the absence of Tet1, other Tet family members can compensate for the activity of Tet1 to regulate neurogenesis.

A recent study from Huangfu’s group has nicely demonstrated that *TET1/2/3* triple knockout human ESCs lose their ability to differentiate into PAX6^+^/SOX1^+^ neuroectoderm cells, which is mainly caused by aberrant hypermethylation at the *PAX6* promoter [14]. In human ESCs, TET1 binding at *PAX6* bivalent promoter leads to the progressive oxidation of 5-methylcytosine. In the absence of 5hmC, de novo DNA methyltransferase DNMT3B anchors at *PAX6* bivalent promoter P0 and induces promoter hypermethylation, which suppresses PAX6 induction and subsequent neuroectoderm differentiation. Targeted demethylation of the *PAX6* P0 promoter by a catalytically inactive Cas9 (dCas9) fused with a TET1 catalytic domain partially restored PAX6 expression and rescued neuron differentiation defects [14]. These results clearly illustrate that TET-mediated hydroxymethylation prevents repressive DNA methylation and ensures key lineage-specific transcription factor expression in neuron differentiation.

### Mesoderm lineage specification

TET2 is ubiquitously expressed in multiple hematopoietic cells, and its mutation is frequently found in hematological malignancies [31, 80, 81]. It has been well documented that TET2 is critical for hematopoiesis. In patients who develop chronic myelomonocytic leukemia and carry *TET2* mutations in CD34^+^ hematopoietic progenitor cells, the TET2-mutated CD34^+^ progenitors preferentially develop into myeloid instead of erythroid cells upon differentiation [82]. Furthermore, it has been shown that single deletion of *Tet2* or double deletion of *Tet2/3* or *Tet1/2* alters T cell, B cell, NKT cell, red blood cell, or mast cell differentiation and maturation [38, 61, 74, 83–85]. For instance, genetic depletion of *Tet2* perturbs the induction of signature cytokine genes upon differentiation of CD4^+^ T cells toward helper T (Th) cells, which is associated with differential enrichment of 5hmC and p300 at the promoters of *Ifng* and *Il17* [38]. In contrast, *Tet1/Tet2* or *Tet2/Tet3 *double knockout mice contain less regulatory T (Treg) cells in the spleen. It has been further shown that Tet proteins can demethylate the conserved noncoding sequences (CNS) in *Foxp3* loci to maintain the expression of Foxp3 [83, 86]. One of the *Foxp3* CNS functions as a super enhancer and docking site for chromatin organizer Satb1 binding [87].

Moreover, TET dioxygenases have also been implicated to play crucial roles during cardiomyocytes and skeletal myoblast differentiation [10, 51, 88]. Knockdown of *Tet2* in undifferentiated C2C12 myoblasts significantly reduces the induction of myoblast differentiation-associated genes *Myog* and *myoM* with elevated methylation at their promoters [88]. Additionally, TET2 is shown to control the expression of contractile genes, such as *SRF, MYOCD*, *and MYH11*, in human coronary artery smooth muscle cells [51]. Similarly, loss of gene expression in smooth muscle cells is correlated with a significant decrease in TET2 binding and 5hmC levels at the promoters of corresponding genes. In cardiomyocyte differentiation, *Tet2* knockdown deregulates a large number of genes associated with heart development and contraction. In particular, induction of the key cardiac gene *Myh7* is suppressed upon cardiomyocyte differentiation in *Tet2* knockdown cells, presumably due to the aberrant methylation at its enhancer [10].

### Endoderm lineage specification

To date, functional analyses of TETs and 5hmC in the context of endodermal lineage specification are still limited. In the intestine, Tet1 is highly expressed in intestinal stem cells and positively regulates the expression of Wnt target genes such as *Axin2* and *Lgr5* [25]. In a model of hepatocyte differentiation from the human hepatic progenitor HepaRG cells, Hernandez-Vargas and colleagues demonstrated that TET1 dioxygenase is necessary for *HNF4A* promoter P1 demethylation and activation upon hepatocyte differentiation [89]. They found that TET1 binds to the P1 locus via the pioneer transcription factor FOXA2, which is required for the establishment of the hepatocyte program [89]. Additionally, to understand the precise role of TET-mediated hydroxymethylation during pancreatic lineage progression, we recently characterized each lineage intermediate, including definitive endoderm, primitive gut tube, posterior foregut, and pancreatic progenitors, in great detail during their stepwise differentiation from human ESCs [28]. We developed genome-wide maps for each stage, encompassing 5mC/5hmC, gene expression, and chromatin architecture/accessibility. We identified that 5hmC is positively correlated with enhancer activities and chromatin accessibility during pancreatic differentiation [28], and further discovered that TET dioxygenases promoted pancreatic endocrine differentiation but had less effect on the early endoderm formation (unpublished results).

## Conclusion and perspectives

In summary, TETs and TET-mediated 5hmC are dynamically redistributed in lineage descendants during development. A substantial number of studies have provided data supporting the role of TET proteins in the establishment of cell identity during differentiation. TET family members alter lineage specification as epigenetic modifiers through their dioxygenase activity to convert 5mC to oxidized methylcytosine. They modulate DNA methylation/hydroxymethylation balance at promoters and enhancers of cell fate-determining genes to further increase chromatin accessibility and facilitate gene transcription in lineage commitment. Strikingly, TET dioxygenases participate in the establishment and/or maintenance of bivalent domains of many differentiation-associated genes, and thusly ensure developmental plasticity. However, direct connections between TET dioxygenases and chromatin architecture are still not clear. Although 5hmC is enriched at distal regulatory elements, how the poised and active enhancer states are influenced by TETs remains elusive. It will be of great interest to identify whether oxidized methylcytosines can act as docking sites and recruit epigenetic readers to modulate histone modifications. Further studies aimed at unraveling the precise role of TET dioxygenases in the control of epigenetic machinery and gene regulation will contribute to the knowledge of how lineage specification is precisely regulated during development.

## Abbreviations

TET: ten-eleven translocation protein
5mC: 5-methylcytosine
5hmC: 5-hydroxymethylcytosine
5fC: 5-formylcytosine
5caC: 5-carboxylcytosine
DNMT: DNA methyltransferase
TDG: thymine DNA glycosylase
BER: base excision repair
DSBH: double-stranded β-helix
CXXC: cysteine-X-X-cysteine
ESCs: embryonic stem cells
NPCs: neural progenitor cells
DMVs: DNA methylation valleys
PRC2: Polycomb Repressive Complex 2
OGT: O-linked *β*-*N*-acetylglucosamine transferase
DMOG: dimethyloxalylglycine
H3K27ac: acetylation of histone H3 at lysine 27
H3K4me1: monomethylation of histone H3 at lysine 4
H3K4me3: trimethylation of histone H3 at lysine 4
H3K27me3: trimethylation of histone H3 at lysine 27
